# Transcriptome sequencing and microRNA–mRNA regulatory network construction in the lens from a Na_2_SeO_3_-induced Sprague Dawley rat cataract model

**DOI:** 10.1186/s12886-023-03202-x

**Published:** 2023-11-16

**Authors:** Rui Fang, Pei-Lin Yue, Hai-Long Li, Xue-Fei Ding, Yu-Xuan Jia, Zhao-Chuan Liu, Hong-Gang Zhou, Xu-Dong Song

**Affiliations:** 1 Beijing Tongren Hospital, Beijing, 100730 China; 2 Capital Medical University, Beijing, 100730 China; 3 The State Key Laboratory of Medicinal Chemical Biology, College of Pharmacy and Key Laboratory of Molecular Drug Research, Nankai University, Tianjin, 300071 China; 4 Beijing Tongren Eye Center, Beijing, China; 5 Beijing, Ophthalmology&Visual Sciences Key Lab, Beijing, China

**Keywords:** mRNA, Cataract, microRNA, Network, Rat, Len

## Abstract

**Background:**

A sight-threatening, cataract is a common degenerative disease of the ocular lens. This study aimed to explore the regulatory mechanism of age-related cataract (ARC) formation and progression.

**Methods:**

Cataracts in Sprague Dawley rats were induced by adopting the method that injected selenite subcutaneously in the nape. We performed high-throughput RNA sequencing technology to identify the mRNA and microRNA(miRNA) expression profiles of the capsular membrane of the lens from Na_2_SeO_3_-induced and saline-injected Sprague Dawley rats. Gene Ontology (GO) and Kyoto Encyclopedia of Genes and Genomes (KEGG) pathway analyses were carried out to forecast the regulatory and functional role of mRNAs in cataracts by DAVID and Metascape. The protein–protein interaction(PPI) network of differentially expressed mRNA(DEmRNAs) was built via the STRING. Target miRNAs of hub genes were predicted by miRBD and TargetScan. Furthermore, differentially expressed miRNA(DEmiRNAs) were selected as hub genes’ targets, validated by quantitative real-time polymerase chain reaction(qRT-PCR), and a DEmiRNA-DEmRNA regulatory network was constructed via Cytoscape.

**Result:**

In total, 329 DEmRNAs including 40 upregulated and 289 downregulated genes were identified. Forty seven DEmiRNAs including 29 upregulated and 18 downregulated miRNAs were detected. The DEmRNAs are involved in lens development, visual perception, and aging-related biological processes. A protein–protein interaction network including 274 node genes was constructed to explore the interactions of DEmRNAs. Furthermore, a DEmiRNA-DEmRNA regulatory network related to cataracts was constructed, including 8 hub DEmRNAs, and 8 key DEmiRNAs which were confirmed by qRT-PCR analysis.

**Conclusion:**

We identified several differentially expressed genes and established a miRNA-mRNA-regulated network in a Na_2_SeO_3_-induced Sprague Dawley rat cataract model. These results may provide novel insights into the clinical treatment of cataracts, and the hub DEmRNAs and key DEmiRNAs could be potential therapeutic targets for ARC.

**Supplementary Information:**

The online version contains supplementary material available at 10.1186/s12886-023-03202-x.

## Introduction

Age-related cataract(ARC) is a prevalent ocular disorder identified by lens opacification, which is the leading cause of blindness in the world [1, 2]. Based on numerous previous investigations, age and environmental factors such as UV exposure and the use of biofuels in rural areas can increase the risk of cataracts [3]. Surgical removal of lens opacity is the mainstream method for cataracts. However, despite the rapid progress in surgery technology related to cataracts, there is still some postoperative complication [4], which influence patients' quality of life and cause serious social economic load. Furthermore, current effective cataract surgical coverage (ECSC) has failed to meet expectations. Median ECSC among high-income countries was highest at 60.5%, but only 14.8% among low-income countries [5]. Therefore, cataract patients in developing countries find it more difficult to obtain surgical resources timely. At present, there is no effective drug to cure cataracts in the clinic, and its specific pathogenesis still needs to be further studied [6]. Considering the global aging trend, it is of far-reaching significance to further improve the level of clinical prevention and treatment of cataracts, and elucidating the mechanism of cataract development is an important cornerstone to achieving this goal.

Transcripts can be identified by transcriptome sequencing technology effectively, mainly including mRNA and non-coding RNA, such as circular RNA, and microRNA (miRNA) [7]. The miRNA is a class of non-coding single-stranded RNA molecules that are widely found in eukaryotes and are encoded by endogenous genes with a length of about 21–23 nucleotides [8]. MiRNA regulates the expression of target genes by binding to the 3’-untranslated region of target mRNA, promoting mRNA degradation or blocking its translation. Current studies have shown that miRNAs are involved in the biological processes of many diseases including tumors, chronic disease, and infectious disease [9, 10], and they can regulate cell proliferation, differentiation, invasion, apoptosis, and metastasis [11]. In recent years, many researchers have detected the expression of miRNAs in the lens of cataract patients, and a large amount of evidence shows that miRNAs are closely related to the development of cataracts, which regulate protein synthesis by regulating mRNA levels [12, 13]. Microarray analysis revealed several miRNA expression differences in cataract crystalline lens compared with normal lens tissue [14]. A growing body of research now indicates that the miRNA-mRNA axis regulates the activity of lens epithelial cells and is involved in the development of cataracts, indicating their potential as a new candidate therapeutic target [13, 15, 16]. For example, the upregulation of Let-7b microRNA has been observed in cataracts and promotes cataract progression [12]. Furthermore, miR-221 and miR-124 were also dysregulated in cataracts, suggesting that these miRNAs may play an important role in the pathogenesis of cataracts [13, 16]. Therefore, there is an urgent need to identify promising miRNAs for the non-surgical treatment of cataracts due to the vacancy of effective miRNAs for cataracts.

Transcriptome sequencing can obtain nearly all transcripts of a particular organ or tissue of a specific species in a certain state comprehensively and quickly, and conduct perfect analysis to evaluate the differential expression of genes, which yields more informative data than microarrays [7, 17]. However, the number of studies using transcriptome sequencing to reveal potential biomarkers for cataracts is limited. Therefore, to identify potential molecular targets for the non-surgical treatment of cataracts, we analyzed mRNA and miRNA expression profiles of the rat cataract model group and control group. Differentially expressed mRNAs (DEmRNAs) and differentially expressed miRNAs (DEmiRNAs) between the rat cataract model and control were selected to construct the miRNA-mRNA network.

In our study, transcriptional sequencing was used to identify differentially expressed miRNAs and mRNAs in selenite-induced rat cataract models, aiming to investigate their potential in cataract prevention and treatment and provide a basis for improving the level of clinical precaution and therapy strategy of cataracts. In addition, to fully understand the functions of proteins and miRNAs in cells and their interactions, we constructed miRNA-mRNA interaction analysis and protein–protein interaction (PPI) networks. The accuracy of RNA sequencing was verified using reverse transcription-quantitative RT(qPCR) for the selected miRNAs. We hope that the identification of cataract-related key miRNAs can contribute to cataract treatment.

## Materials and methods

### Establishment of the rat model of cataract

All Sprague–Dawley (SD) rats used in this research were purchased from Weitong Lihua Biotechnology (Beijing, China). Healthy 8-day-old SD rat pups were used to establish the cataract model, they were housed along with their mothers for 14 days at 25°C in a barrier environment with a 12/12 h light/dark cycle. All rats were monitored daily for the development of cataracts by slit-lamp. All animal experiments were approved by the Institutional Animal Care and Use Committee (IACUC) of Nankai University (Permit No. 2022-SYDWLL-000550).

**Fig. 1:**
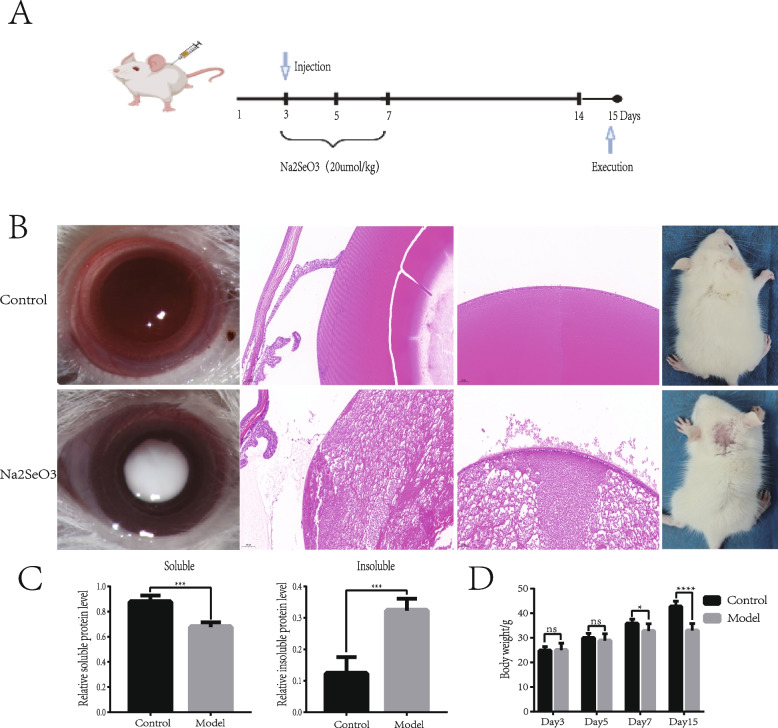
Establishment of the Na_2_SeO_3_-induced Sprague Dawley rat cataract model. **A** The experimental process of Na_2_SeO_3_-induced Sprague Dawley rat cataract model construction. **B** The slit-lamp image and HE staining of lenses in control and Na_2_SeO_3_-induced groups. **C** Soluble and insoluble proteins of lenses were analyzed in control and Na_2_SeO_3_-induced groups (*n* = 3). **D** Body weight was measured before administration and anesthesia (*n* = 10)

Before the experiment, the SD rat pups were split at random into two groups: the control group(rat pups treated with saline) and the Na_2_SeO_3_ group(rat pups treated with sodium selenite at 20μmol/kg body weight). Animals in the Na_2_SeO_3_ group were injected subcutaneously on the nape of the neck with Na_2_SeO_3_ on days 3, 5, and 7, and animals in the control group were treated in the same way with the equivalent amount of saline, essentially as described previously [18]. On the 15th day of the experiment, the rat pups were sacrificed under ketamine anesthesia and the lens was extracted. The diagram of model establishment is shown in Fig. 1A. All experimental operations were following the Guidelines for the Care and Use of Laboratory Animals.

### Soluble and insoluble protein assay

The rat lens was removed from the corneal scleral margin and protein levels were measured using the lens of the right eye. After the lens was cleaned with ice PBS and weighed, it was homogenized in PBS and NaOH with an ultrasound homogenizer and centrifuged at 4℃ at 12,000 G for 25 min. The supernatants obtained from the two solvents were used to detect the concentrations of soluble and insoluble proteins respectively, essentially as described in detail previously [18]. The concentration of soluble protein and insoluble protein was determined by a BCA protein concentration assay kit (Gene-protein link, Beijing, China).

### Haematoxylin and eosin(HE) staining

For a check of the pathological and ultrastructural changes of the lens in each group, the removed eyeballs were fixed with FAS eyeball fixation solution(Gene-protein link, Beijing, China) for 48h. Next, the fixed tissues were dehydrated and embedded in paraffin, cut into 5um thick slices, HE staining was then performed according to standard procedure and observed by a Microscope slide scanner (3D hi-tech/Pannoramic MIDI, Hungary).

### High-throughput RNA sequencing (RNA-Seq)

After extracting total RNA from the capsular membrane of six lenses (three rats from the control group and three rats from the Na_2_SeO_3_ group) using TRIzol reagent (ThermoFisher, USA). RNA quality inspection was performed by an Agilent 2100 RNA Nano 6000 Assay Kit (Agilent Technologies, CA, USA). The Qubit® 2.0 Fluorometer (Life Technologies, CA, USA) was used in the RNA concentration measurement. Sequencing library construction and sequencing were carried out by Beijing Tangtangtianxia Biotechnology Co. Ltd (Beijing, China).

The data from RNA-Seq were first filtered (Cutadapt and Fqtrim) and assessed for quality (FastQC), and then compared to the reference genome (miRDeep and HISAT2). The data source for the reference genome is the Ensembl database (http://asia.ensembl.org).

### Screening of differentially expressed RNAs

We used the edgeR [19] package to analyze the high-throughput sequencing data, genes with adjusted *P*-value < 0.05 and llog2FoldChangel > 1 were selected as DEmiRNAs and DEmRNAs. Volcanic maps and heat maps of DEmiRNAs and DEmRNAs were generated using the Tutu Cloud Platform (http://www.cloudtutu.com/#/index).

### GO and KEGG pathway enrichment analysis

Gene Ontology (GO) and Kyoto Encyclopedia of Genes and Genomes (KEGG) pathway enrichment analysis were performed for DEmRNAs, using the online Database for Annotation, Visualization and Integrated Discovery (DAVID) [20] (https://david.ncifcrf.gov/) and Metascape [21] (http://metascape.org), which is a powerful tool for gene function annotation analysis. *P* value < 0.05 as the cutoff criterion was considered statistically significant.

### Protein–protein interaction(PPI)network analysis

Protein interaction network analysis is important for understanding protein function and relationship. The PPI network of upregulated and down-regulated DEGs-encoded proteins was demonstrated by the STRING website [22] (version 11.5, https://cn.string-db.org), with a search limited to “Rattus norvegicus” and a score > 0.4 corresponding to medium confidence interaction as significant. Network construction and analyses were performed by Cytoscape [23] software(version 3.9.1). furthermore, the hub genes of the PPI network analysis were performed for DEmRNAs in the modules by Hubba (version 2.5.4), and a *P* value < 0.05 was considered to be significant.

### Construction of a miRNA-mRNA Regulatory Network

The miRBD(https://mirdb.org/) and TargetScan(https://www.targetscan.org/vert_80/) websites were used in the target miRNA prediction of the hub genes. Venn diagrams of the overlap between target miRNAs and DEmiRNAs were generated using Bioinformatics & Evolutionary Genomics (http://bioinformatics.psb.ugent.be/webtools/Venn/). Based on the overlap, Cytoscape software(version 3.9.1) was used to visualize the miRNA-mRNA regulatory network.

### Cell culture and treatment

The human lens epithelial cell line (HLEC) SRA01/04 used in this study was purchased from the American Type Culture Collection (ATCC) and validated by short tandem repeat (STR) analysis (Genetic Testing Biotechnology, Suzhou, China). SRA01/04 cells were cultured in DMEM medium (Gibco, C11995500BT) with 10% fetal bovine serum(Gibco, USA). All cells were maintained at 37 °C in an incubator with 5% CO_2_. The cells were treated with 200 μM H_2_O_2_ (Millipore, Bradford, MA, USA) for 24 h to establish an ARC cell model.

### qRT-PCR verification of selected miRNAs

After HLECs were treated with H_2_O_2_ for 24 h, the culture medium was discarded, washed with PBS, and RNA was extracted with TRIzol reagent (ThermoFisher, USA). Total RNA was extracted according to the instructions of the manufacturer and concentration was determined using Nanodrop One (ThermoFisher, USA). A total of 1 ug of total RNA was converted into cDNA using one-step RT-gDNA digestion SuperMix (Yeasen, China). Universal Blue qPCR SYBR Green Master Mix (Yeasen, China) was used to perform qRT PCR. The PCR primers of selected genes were synthesized by Sangon Biotech (Shanghai, China), and detailed information on primers is provided in Table 1. Relative expressions of target genes were calculated according to the 2^−ΔΔCt^ method. All the PCR reactions were performed in three biological repetitions.

**Table1:** Primer for Quantitative real-time PCR (qRT-PCR)

Gene names	Primer sequence (5'-3')
miR-206-3p	Forward-GCCGAGTGGAATGTAAGGAA
miR-493-5p	Forward-GCCGAGTTGTACATGGTAGG
miR-493-3p	Forward-GCCGAGTGAAGGTCTACTG
miR-193b-3p	Forward-GCCGAGAACTGGCCTACAAA
miR-200a-3p	Forward-GCCGAGTAACACTGTCTGGT
miR-29a-3p	Forward-GCCGAGTAGCACCATCTGAA
miR-29b-3p	Forward-GCCGAGTAGCACCATTTGAAA
miR-29c-3p	Forward-GCCGAGTAGCACCATTTGAA
U6	Forward-TGCGGGTGCTCGCTTCGGCAGC
Reverse	TATCCAGTGCAGGGTCCG

### Statistics analysis

Data were presented as the mean ± standard deviation (SD). Statistical analysis was performed using GraphPad Prism7.0 (GraphPad Software, CA, USA). Student’s t-test was used for comparison, differences were considered significant at *p* < 0.05. Statistical differences were indicated as follows: * *p* < 0.05, ** *p* < 0.01, *** *p* < 0.001, and **** *p* < 0.0001 compared to the control. All assays were carried out three times or more.

## Result

### Establishment and evaluation of Na_2_SeO_3_-induced cataracts in rats

As shown in Fig. 1A, after three times of subcutaneous injections, the eyes of the rat pups were photographed on the 15th day of the experiment to observe the opacity of the lenses. The rat pups were then sacrificed and their eyeballs were removed for further analysis. The lenses of rat pups in the normal control group showed complete transparency, while all Na_2_SeO_3_-treated rats showed cataracts with lenses opacity. Typical images of the cataract group and the control group are shown in Fig. 1B. H&E staining was used to evaluate the histology of the rat eye samples, and it was found that the lens epithelium and lens fibers remained intact and well-organized without obvious pathological changes in the normal control group. Visibly, histological analysis revealed diffuse cataract changes in the lens in the Na_2_SeO_3_ group, including mussy and loose arrangement of lens epithelial cells, and degeneration/liquefaction of lens fibers (Fig. 1B). We detected the levels of soluble and insoluble proteins in the lenses of rats in the two groups and found that the level of soluble protein in the model group was notably descended, while the level of insoluble protein was markedly increased (Fig. 1C). We found that the body weight of the experimental group was remarkably decreased compared with that of the healthy control group on days 7 and 15 (day7:model group 32.79 ± 2.893g, control group 35.73 ± 1.837g, *p* = 0.018; day15:model group 32.92 ± 2.814g, control group 42.68 ± 2.21g, *p* < 0.0001), indicating the developmental retardation of Na_2_SeO_3_-treated rats (Fig. 1D). Moreover, as demonstrated in Fig. 1B, animals in the control group had intact hair, while there was baldness at the injection site of those in the model group.

### Analysis of high-throughput RNA sequencing results

**Fig. 2:**
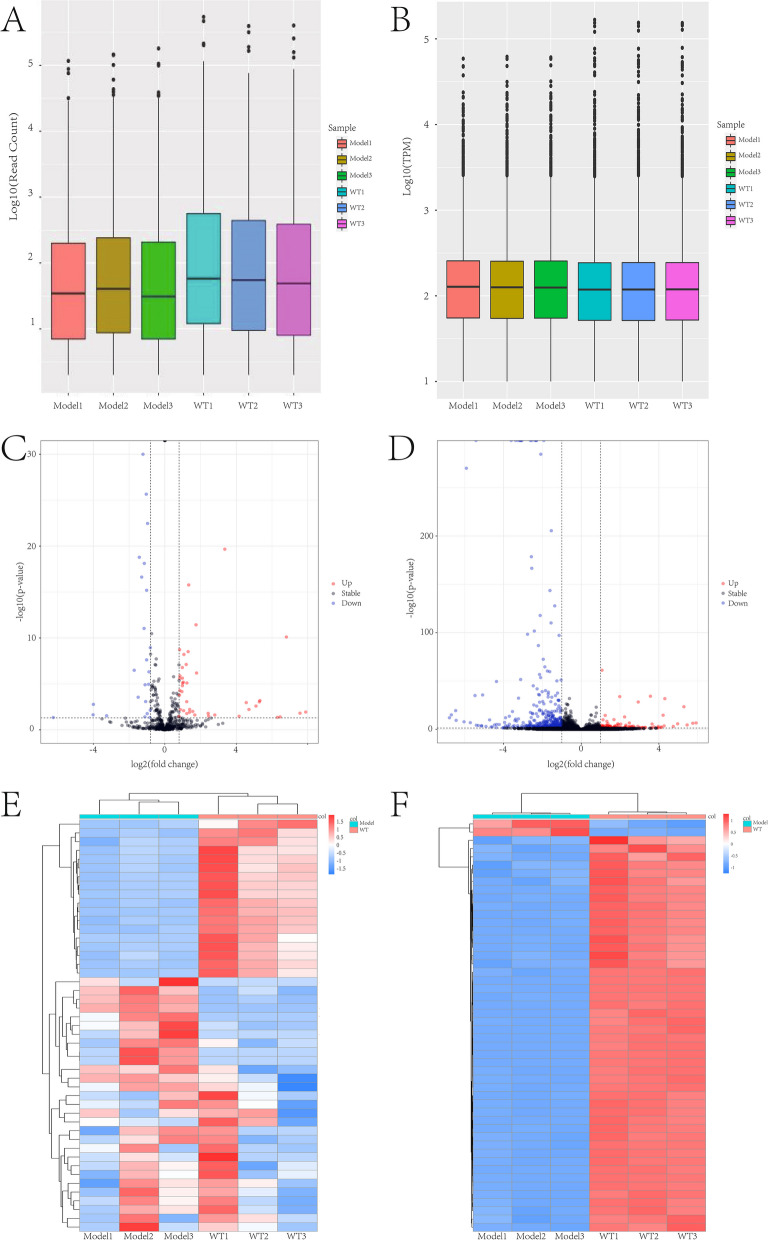
Identification of the significant expression changes of RNAs in cataract rat models. **A** The distribution of the total expression of the miRNA in six lenses. **B** The distribution of the total expression of the mRNAs in six lenses. **C** Volcano plots of the DEmiRNAs (red, upregulated; blue, down-regulated). **D** Volcano plots of the DEmRNAs (red, upregulated; blue, down-regulated). **E** Heatmaps for DEmiRNAs (red, upregulated; blue, down-regulated). **F** Heatmaps for DEmRNA (red, upregulated; blue, down-regulated)

The RNA-Seq results revealed the expression profiles of differentially expressed miRNAs and mRNAs between the lenses of cataractous rats and control rats (including three experimental animals and three control animals)(GEO Submission: GSE226274) (www.ncbi.nlm.nih.gov/geo). With *P*-value < 0.05 and llog2FoldChangel > 1 as thresholds, 47 differentially expressed miRNAs and 329 differentially expressed mRNAs were found in miRNA and mRNA expression profiles. Information of Raw Data (FASTQ) about reading counts and Q30% etc. are demonstrated in Table 2. Compared with the normal lens, 29 miRNAs and 40 mRNAs were up-regulated and 18 miRNAs and 289 mRNAs were down-regulated in the cataractous lens. The normalized miRNAs/mRNAs dataset distributions of each group were basically the same (Fig. 2A and B). A volcano plot of the expressions of miRNA and mRNA shows the up-regulated and down-regulated miRNAs and mRNAs (Fig. 2C and D). Heat map clustering was used to analyze miRNAs and mRNAs expression levels (Fig. 2E and F).

**Table 2:** Information of Raw Data(FASTQ) about RNA-Seq

	Sample	Raw Reads	Bases(Gp)	GC(%)	Q20(%)	Q30(%)
miRNA	Model1	5,793,340	0.29	52.04	98.38	95.3
	Model2	7,431,649	0.372	51.88	98.95	96.58
	Model3	7,463,788	0.373	51.69	98.86	96.26
	WT1	7,128,168	0.356	50.78	98.91	96.32
	WT2	7,881,554	0.394	50.65	99.08	97
	WT3	7,056,397	0.353	50.45	98.92	96.39
mRNA	Model1	117,332,702	17.717	46.955	97.23	93.115
	Model2	121,218,758	18.304	46.26	97.06	92.685
	Model3	111,458,008	16.83	46.34	97.02	92.63
	WT1	105,396,088	15.915	47.785	96.915	92.345
	WT2	87,462,256	13.207	47.85	96.935	92.55
	WT3	118,907,686	17.955	47.325	96.945	92.28

### Functional enrichment analysis and protein–protein interaction(PPI) analysis

**Fig. 3:**
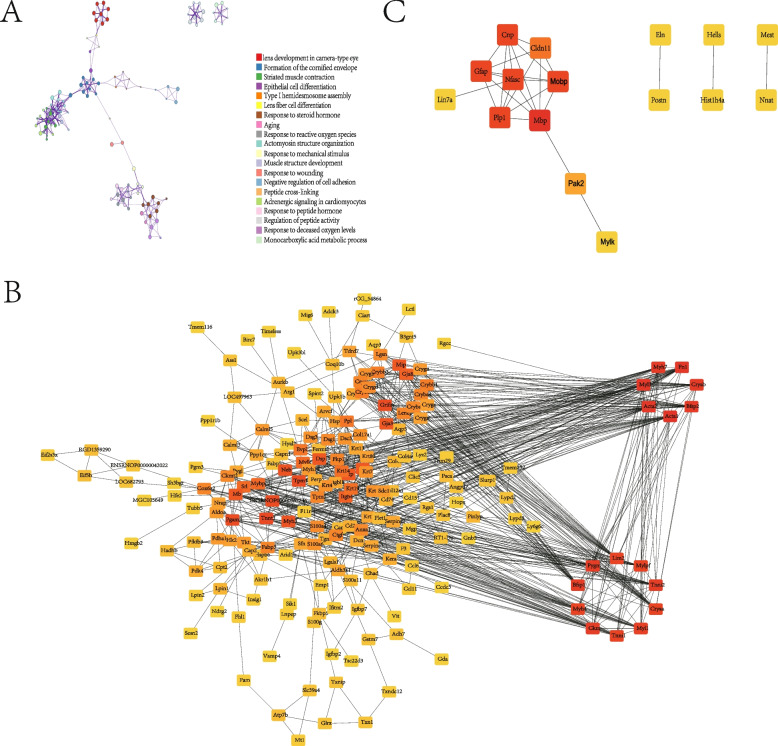
Three types of networks. **A** Top 20 clusters and their representative enriched terms (colored by cluster ID). **B** The PPI network of down-related DEmRNAs by the online database STRING. **C** The PPI network of up-related DEmRNAs by the online database STRING

Three hundred and twenty-nine DEmRNAs were analyzed by the online software Metascape. The top 20 GO and KEGG pathway terms of 329 mRNAs are shown in Fig. 3A, some enriched functions and pathways were related to lens development and aging. The GO functional analysis of the differentially expressed mRNA was categorized into the following three parts: biological process (BP), molecular function (MF), and cell component (CC), and terms with p values less than 0.05 were listed in Figure S1A. Among the involved biological processes, lens development in the camera-type eye, visual perception, muscle contraction, camera-type eye development, and intermediate filament organization, were the most important terms related to the dysregulated mRNAs. Regarding CC, DEmRNAs were significantly enriched in the intermediate filament. The molecular function of DEmRNAs was significantly enriched in the structural constituent of the eye lens and structural molecule activity. Besides, adrenergic signaling in cardiomyocytes, cardiac muscle contraction, and hypertrophic cardiomyopathy were the most notably enriched KEGG pathway terms (Figure S1 B).

The up-regulated and down-regulated DEmRNAs were analyzed using the STRING online database, and the PPI network was constructed. The results were downloaded and analyzed using Cytoscape software (Fig. 3 B and C). The top 5 hub genes of up-regulated DEmRNAs and 10 hub genes of down-regulated DEmRNAs were screened according to their degree values, and the identified hub genes were Mbp, Nfasc, Gfap, Cnp, Plp1, Acta1, Acta2, Fn1, Myl3, Cryab, Myh7, Bfsp2, Ckm, Pygm and Bfsp1, as shown in Table 3.

**Table 3:** The degree values of the key genes

	Gene symbol	Gene description	log2FC	Degree
Up-regulated DEmRNAs	Mbp	myelin basic protein	4.31	7
	Nfasc	neurofascin	2.57	6
	Gfap	glial fibrillary acidic protein	3.56	6
	Cnp	2',3'-cyclic nucleotide 3' phosphodiesterase	1.32	6
	Plp1	proteolipid protein 1	2.94	6
Down-regulated DEmRNAs	Acta1	actin, alpha 1, skeletal muscle	-1.22	34
	Fn1	fibronectin 1	-1.12	29
	Myl3	myosin light chain 3	-2.23	26
	Cryab	crystallin, alpha B	-3.09	25
	Myh7	myosin heavy chain 7	-1.52	25
	Bfsp2	beaded filament structural protein 2	-2.1	25
	Ckm	creatine kinase, M-type	-1.15	24
	Pygm	glycogen phosphorylase, muscle associated	-1.28	24
	Bfsp1	beaded filament structural protein 1	-2.57	24
	Myh4	myosin heavy chain 4	-1.53	23

*FC* fold change

### Construction of the DEmiRNA-mRNA regulatory network

**Fig. 4:**
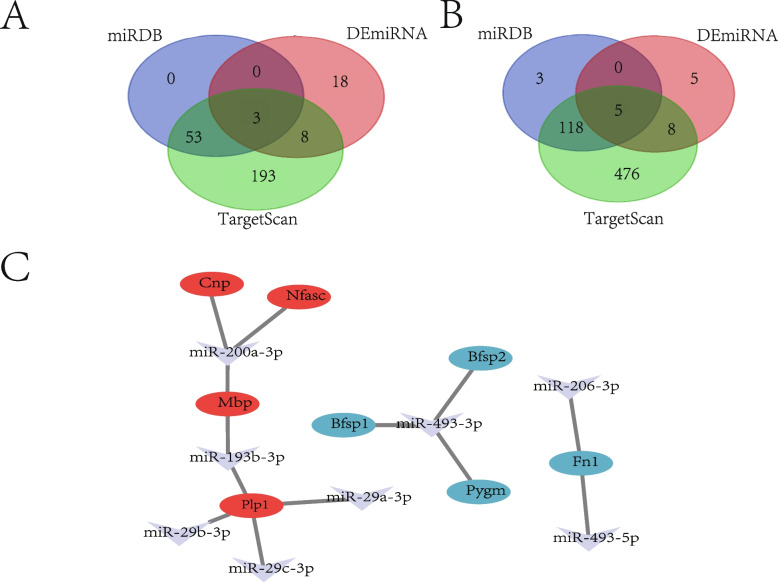
Construction of miRNA-mRNA regulatory network. **A** Venn diagram of DEmiRNAs and predicted miRNAs of down-related mRNA by Targetscan/miRDB. **B** Venn diagram of DEmiRNAs and predicted miRNAs of up-related mRNA by Targetscan/miRDB. **C** Construction of the miRNA-mRNA regulatory network including 8 miRNAs and 8 mRNAs (red ellipse, up-regulated DEmRNAs; blue ellipse, down-regulated DEmRNAs; V-shape, DEmiRNAs)

Using TargetScan and miRDB to predict the target miRNAs of the up-regulated and down-regulated hub genes, the results of TargetScan were visualized in Figure S2A and S2B. Based on the negative regulation relationship between miRNAs and mRNAs, the intersection of predicted miRNAs obtained from the above analysis and differentially expressed miRNAs was taken to obtain 8 key miRNAs. For the target miRNAs of 10 down-regulated hub genes and up-regulated DEmiRNAs, the intersection set was taken to obtain 3 key miRNAs including miR-206-3p, miR-493-5p, and miR-493-3p (Fig. 4A). The overlap between the targets miRNAs of 5 up-regulated hub genes and down-regulated DEmiRNAs was shown in Fig. 4B, including miR-193b-3p, miR-200a-3p, miR-29a-3p, miR-29b-3p, and miR-29c-3p. The regulatory network of miRNA-mRNA was established, as shown in Fig. 4C, involving 8 miRNAs and 8 hub-genes (Bfsp1, Plp1, Cnp, Nfasc, Mbp, Pygm, Bfsp2, and Fn1).

### Verification of differentially expressed genes in the network by qRT-PCR

**Fig. 5:**
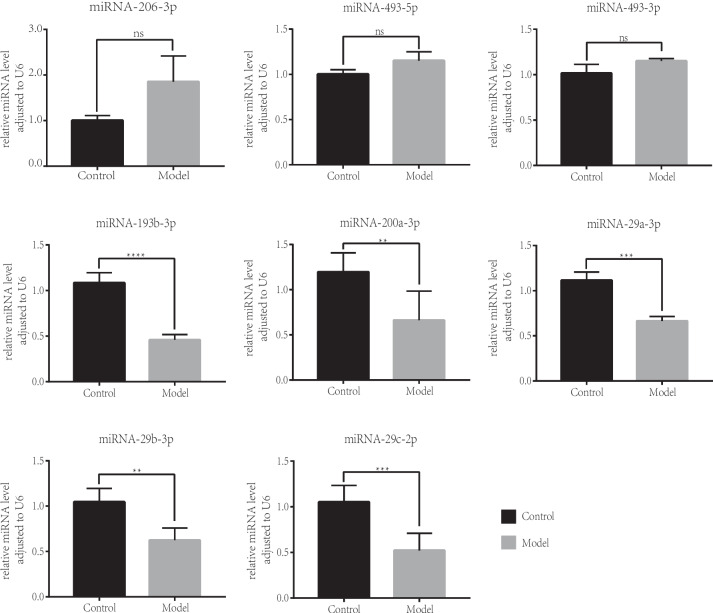
The expression of key DEmiRNAs in H_2_O_2_ induced model group compared with the control group by qPCR (*n* = 3)

To confirm the reliability of our findings, we validated the identified miRNAs of the network by qRT-PCR in vitro model of cataract induced by H_2_O_2_. According to our experimental results, The related expression to U6 of miRNA-206-3p, miRNA-493-5p, and miRNA-493-3p was lightly upregulated with no significance. The relative expression of miRNA-193b-3p, miRNA-200a-3p, miRNA-29a-3p, miRNA-29b-3p, and miRNA-29c-3p were significantly decreased in the H_2_O_2_-induced model group, in agreement with our bioinformatics expression analysis (Fig. 5).

## Discussion

Cataract is a common ophthalmic ailment characterized by clouding of the lens caused by multiple factors that can gradually lead to visual impairment [2]. Although clinicians are currently able to surgically remove cloudy lenses and improve vision of cataract patients, non-surgical options remain attractive considering surgical resources and postoperative complications. However, its complex pathogenesis is a stumbling block in the development of non-surgical therapies, and new biomarkers are needed to advance this work. Over the years, many studies have been devoted to elucidating the pathogenesis of cataracts, and they have found some valuable molecules that, based on their results in preclinical studies, may be promising therapeutic targets [24]. The miRNA is like a rising star among them, which attracts more and more concerns, emerging as a prominent class of gene regulators [25]. Based on existing studies, we found that not only a single miRNA can target multiple target genes, but also multiple miRNAs can regulate the same gene. This complex regulatory network enables miRNA to fully exert its regulatory effect on genes. It can not only regulate the expression of multiple genes through a single miRNA but also control the expression of a certain gene through the combination of multiple miRNAs precisely [8, 26]. However, the role of miRNAs in the occurrence and development of cataracts remains largely unknown and needs to be clarified by further studies. The advance in sequencing technology, such as high-throughput RNA sequencing technology (HST), provides a powerful mining tool for related research.

Na_2_SeO_3_-induced cataract model, an extremely rapid and convenient model of nuclear cataracts produced in young rats [27, 28]. Compared to other cataract models, vesicles formed in selenite cataracts most closely resemble those in human cataracts by confocal microscopy. Selenite cataracts share other similarities with human cataracts, such as increased calcium, insoluble protein, and proteolysis; decreased water-soluble proteins and reduced glutathione [28, 29]. There are some differences between selenium cataracts and human cataracts, so careful consideration is needed when extrapolating selenium cataracts results to humans. Accordingly, we determined miRNA and mRNA expression profiling between paired Na_2_SeO_3_-induced rat cataract model and control animals using HTS technology, aiming to distinguish deregulated RNAs in cataract lenses and try to discover its potential and effect as a non-surgical treatment for cataract. We also conducted further verification in human lens epithelial cells.

The competing endogenous RNA (ceRNA) is a novel mechanism for exploring the interactions of RNA at the post-transcriptional level by competing for shared microRNA response elements (MREs) [30]. For example, mRNA, miRNA, and circRNA were known as RNA interaction molecules, which have crucial biological significance [31, 32]. As the center of the ceRNA regulatory network, miRNA acts as the downstream of circRNA/lncRNA to directly regulate the expression and translation of target genes [8]. The gain or loss of function of individual miRNAs can change the expression levels of hundreds of proteins [26], which means that a single miRNA can alter multiple pathophysiological phenotypes at the same time regardless of cataractogenic etiology. Therefore, the manipulation of miRNA expression is a promising strategy that has preventive and therapeutic effects on cataracts. In this study, a miRNA-mRNA network was constructed which contains miR-206-3p, miR-493-5p, miR-493-3p, miR-193b-3p, miR-200a-3p, miR-29a-3p, miR-29b-3p, miR-29c-3p, Bfsp1, Plp1, Cnp, Nfasc, Mbp, Pygm, Bfsp2 and Fn1, whose dysregulation may be associated with the cataract pathogenesis.

Fn1(fibronectin-1) is a member of the glycoprotein family that has been shown to play an important role in cancer metastasis [33], osteogenic differentiation [34], and epithelial-mesenchymal transformation(EMT) [35]. Posterior capsule opacification (PCO) is a complication after cataract surgery, particularly in children. Migration and abnormal differentiation of lens epithelial cells (LECs) in the form of EMT are the major biological processes associated with PCO. As a major marker of typical EMT, the elevation of fibronectin was evident in TGFβ2-stimulated LECs, contributing to PCO [36]. In the Na_2_SeO_3_-induced cataract group, the level of Fn1 was decreased, with a marked difference from the control group, indicating Fn1 participates in the process of cataract. Beaded filaments are the major cytoskeletal element of the eye lens and they are essential to the optical properties of the eye lens [37]. Among them, BFSP2 (phakinin or CP49) and BFSP1 (filensin) assemble into lens-specific beaded intermediate filaments, which are one of the main cytoskeletal structures in the lens fiber [38, 39]. BFSP1 and BFSP2 have been confirmed to be genes associated with autosomal dominant cataracts [40, 41]. Moreover, the absence of BFSP2 or BFSP1 causes the disappearance of beaded intermediate filaments and changes the morphology of lens fiber cells [42]. Both of them decreased in the model group and considering the arrangement disorder, degeneration, and liquefaction of fiber cells in the lens of the Na_2_SeO_3_-induced cataract group, suggested that they were involved in the formation of cataracts.

MiRNA-29a, miRNA-29b, and miRNA-29c belong to the miRNA-29 family. Studies have shown that miR-29b can inhibit the deposition of extracellular matrix and increase cell viability under chronic oxidative stress conditions, and ameliorate oxidative damage [43]. The decreased expression of miRNA-29a and miRNA-29c has been proven to exert function in the apoptosis of LECs during diabetic cataract formation by targeting the BCL2-modifying factor [44]. Moreover, the cytoskeleton remodeling genes tropomyosin (Tm) 1 α and 2 β were targets of miR-29c [45], which have been implicated in the pathophysiology of fibrosis in the lens [46]. Dunmire et al. identified 110 miRNAs in the aqueous humor of cataract patients, and miR-202, miR-193b, miR-135a, miR-365, and miR-376a were the top 5 abundant miRNAs [47]. Studies have shown that the expression of microRNA-200a [48], microRNA-493 [49], and miR-206 [50] were related to cell apoptosis, senescence, and dysfunction, which may be involved in cataract pathogenesis. Therefore, we concluded that miR-206, miR-493, miR-193b, miR-200a, and miR-29 may play important roles in a variety of biological processes by regulating the expression of target genes with different functions (Bfsp1, Plp1, Cnp, Nfasc, Mbp, Pygm, Bfsp2, and Fn1), which plays an important place in the pathogenesis network of cataract.

If a miRNA acts on target genes and regulates the development of cataracts, we can try to insert its pre-miRNA or sponge sequence into viral vectors to correct the pathogenic genes of cataracts by manipulating miRNA expression, which may provide a new approach for the treatment of cataracts. At present, small nucleic acid drug research is in full swing and there are many applications in eye diseases. Our research results show that they provide a new idea for the drug treatment of cataracts, and they may become effective anti-cataract drugs in the future. However, there are still some limitations in this study. The specific mechanism of these selected miRNAs in cataracts is not yet clear, and their regulatory mechanism needs to be clarified by further in vitro and in vivo experiments.

## Conclusion

In summary, we constructed a miRNA-mRNA network in a cataract rat model, which helps understand the molecular mechanism of cataracts. Some important genes and pathways related to cataracts were identified, and these results may suggest some guidance for potential effective therapy.

### Supplementary Information

**Additional file 1.** 

## Data Availability

The datasets generated and analyzed during the current study are stored in Gene Expression Omnibus (GEO) (https://www.ncbi.nlm.nih.gov/geo/query/acc.cgi?acc=GSE226274) and available from the corresponding author on reasonable request.
