# Supplementary material for: Transcriptome sequencing and microRNA–mRNA regulatory network construction in the lens from a Na2SeO3-induced Sprague Dawley rat cataract model
Source: BMC Ophthalmol. 2023 Nov 16;23:461. doi: 10.1186/s12886-023-03202-x (PMC10652440; doi:10.1186/s12886-023-03202-x)
Supplement: Supplementary file 1 — Additional file 1. [file 12886_2023_3202_MOESM1_ESM.pdf]

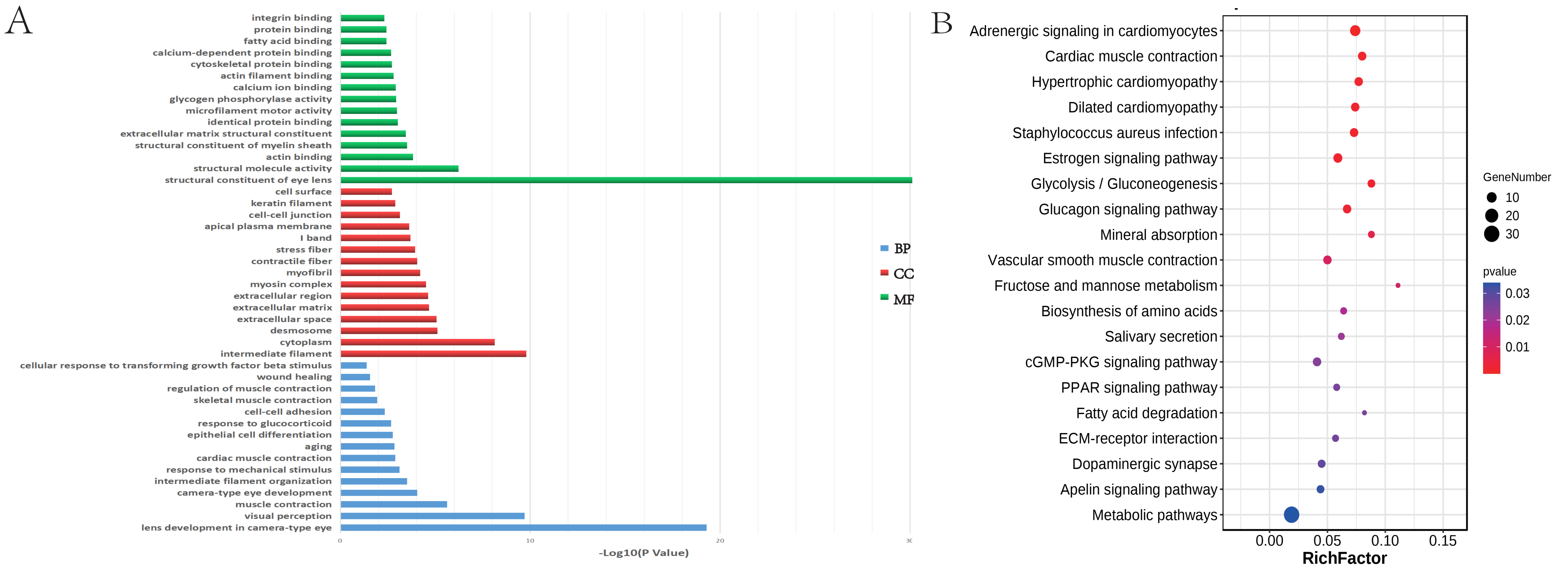
A
integrin binding
protein binding
fatty acid binding
calcium-dependent protein binding
cytoskeletal protein binding
actin filament binding
calcium ion binding
glycogen phosphorylase activity
microfilament motor activity
identical protein binding
extracellular matrix structural constituent
structural constituent of myelin sheath
actin binding
structural molecule activity
structural constituent of eye lens
cell surface
keratin filament
cell-cell junction
apical plasma membrane
I band
stress fiber
contractile fiber
myofibril
myosin complex
extracellular region
extracellular matrix
extracellular space
desmosome
cytoplasm
intermediate filament
cellular response to transforming growth factor beta stimulus
wound healing
regulation of muscle contraction
skeletal muscle contraction
cell-cell adhesion
response to glucocorticoid
epithelial cell differentiation
aging
cardiac muscle contraction
response to mechanical stimulus
intermediate filament organization
camera-type eye development
muscle contraction
visual perception
lens development in camera-type eye
BP
CC
MF
0
10
20
30
-Log10(P Value)
B
Adrenergic signaling in cardiomyocytes
Cardiac muscle contraction
Hypertrophic cardiomyopathy
Dilated cardiomyopathy
Staphylococcus aureus infection
Estrogen signaling pathway
Glycolysis / Gluconeogenesis
Glucagon signaling pathway
Mineral absorption
Vascular smooth muscle contraction
Fructose and mannose metabolism
Biosynthesis of amino acids
Salivary secretion
cGMP-PKG signaling pathway
PPAR signaling pathway
Fatty acid degradation
ECM-receptor interaction
Dopaminergic synapse
Apelin signaling pathway
Metabolic pathways
0.00
0.05
0.10
0.15
RichFactor
GeneNumber
10
20
30
pvalue
0.03
0.02
0.01

Figure-S1. Significantlly enriched GO terms and KEGG pathways of common DEmRNAs.

(A) GO terms(green, Biological process (BP); red, Cell composition (CC); blue, Molecular function (MF)).

(B) Kyoto Encyclopedia of Genes and Genomes (KEGG) pathways.

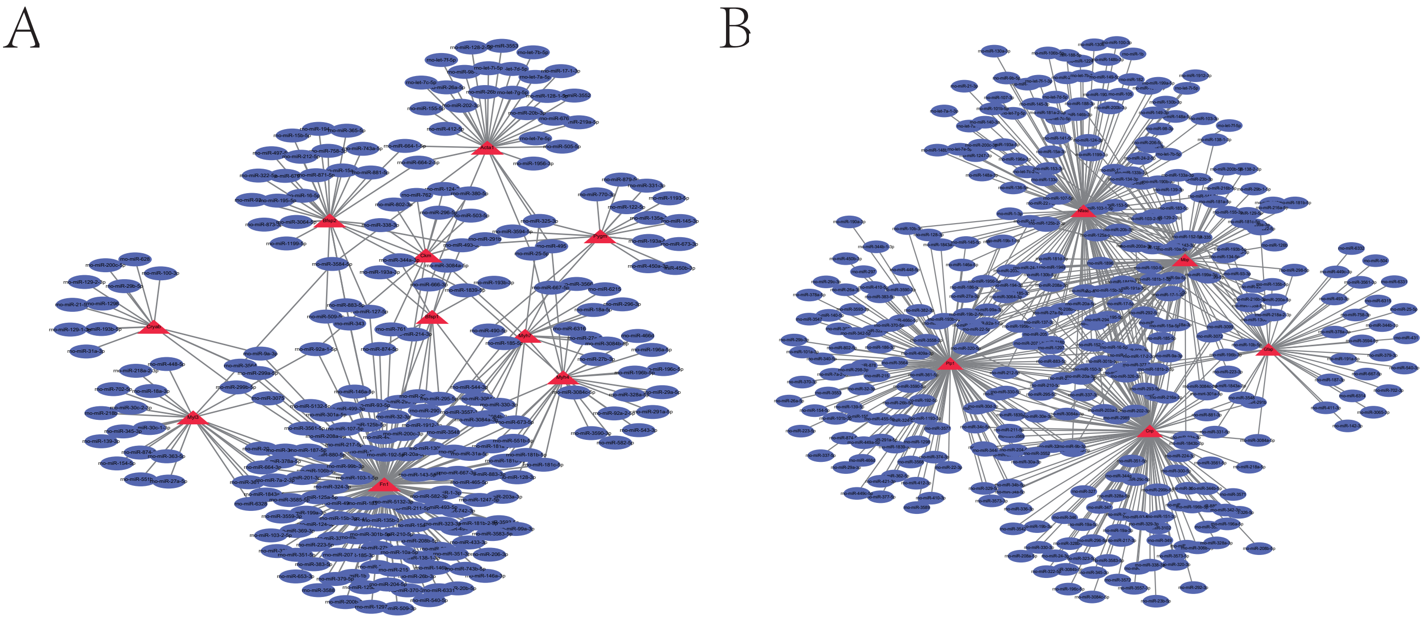
A
B

Figure-S2. Predicted miRNAs of hub genes by Targetscan.

(A) Predicted miRNAs of hub genes in down-regulated mRNA.

(B) Predicted miRNAs of hub genes in up-regulated mRNA(part).
